# Paternal Sperm *Gnas*‐ICR Epigenetic Programming Contributes to PPP‐Like Phenotypes in Female Offspring

**DOI:** 10.1002/advs.76434

**Published:** 2026-07-28

**Authors:** Jing Huang, Lu Chen, Tiancheng Wu, Yating Li, Hui Wang

**Affiliations:** ^1^ Department of Gynaecology and Obstetrics Zhongnan Hospital of Wuhan University Wuhan China; ^2^ Department of Otorhinolaryngology Head and Neck Surgery Zhongnan Hospital of Wuhan University Wuhan China; ^3^ Provincial Key Laboratory of Developmentally Originated Disease Wuhan China; ^4^ Department of Pharmacology Basic Medical School of Wuhan University Wuhan China

**Keywords:** estrogen synthesis, Gnas‐ICR, imprinted genes, paternal exposure, peripheral precocious puberty, sperm reprogramming

## Abstract

The incidence of peripheral precocious puberty (PPP) in females has been rising steadily, emerging as a significant public health concern. However, the paternal developmental origins of PPP remain poorly understood. Using a rat model, we found that paternal preconception caffeine exposure (PPCE) induced PPP‐like phenotypes in female offspring and showed paternal‐line persistence to the F2 generation. Mechanistic analyses showed that PPCE induced a paternal glucocorticoid‐elevated state and was associated with hypermethylation of the *Gnas* imprinting control region (ICR) in sperm. In offspring ovaries, this alteration was paralleled by increased *Gnas*‐ICR methylation, reduced Nespas expression, increased Gnas expression, activation of the cAMP/PKA/CREB pathway and enhanced estrogen biosynthesis. Ovarian Gnas gain‐ and loss‐of‐function experiments supported a functional contribution of Gnas dysregulation to steroidogenesis and PPP‐like phenotypes, while paternal GR antagonism attenuated sperm/ovarian methylation changes and offspring phenotypes. Exploratory human samples suggested a weak and method‐sensitive association between plasma cortisol levels and sperm *Gnas*‐ICR methylation. These findings support a paternal glucocorticoid–associated sperm *Gnas*‐ICR mechanism that may contribute to offspring ovarian endocrine programming. Together, these findings provide a preclinical framework for understanding how paternal preconception endocrine status may shape offspring reproductive development through sperm‐associated epigenetic programming.

## Graphical abstract

A paternal glucocorticoid–sperm Gnas‐ICR axis is linked to ovarian endocrine programming in female offspring. Paternal preconception caffeine exposure elevates corticosterone, promotes sperm Gnas‐ICR hypermethylation, and is associated with ovarian Gnas upregulation, cAMP/PKA/CREB–StAR activation, enhanced estradiol synthesis, and PPP‐like phenotypes across the paternal line.

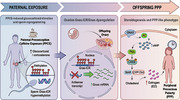

## 1 Background

The initiation of puberty represents a precisely regulated developmental milestone, reflecting the coordinated maturation of the endocrine, metabolic, and reproductive systems. Disruptions in this process—particularly the occurrence of precocious puberty—are not only associated with long‐term metabolic disorders and reproductive dysfunction but also confer an increased risk of hormone‐dependent diseases [1, 2]. In clinical classification, peripheral precocious puberty (PPP) is defined by gonadotropin‐independent overproduction of sex steroids, typically driven by autonomous steroidogenesis in the gonads [2, 3, 4]. Although PPP accounts for approximately 50%–60% of all precocious puberty cases and exhibits a significantly higher prevalence in females compared to males [5, 6, 7], current understanding of its developmental origins and intergenerational regulatory mechanisms remains limited.

The traditional “Developmental Origins of Health and Disease (DOHaD)” paradigm has long emphasized the influence of the maternal prenatal environment on offspring developmental trajectories [8, 9, 10]. However, accumulating evidence in recent years has prompted a paradigm shift: paternal environmental exposures prior to conception—such as nutritional imbalances, psychological stress, and exposure to environmental endocrine‐disrupting chemicals—can alter the metabolic and neurodevelopmental phenotypes of offspring through sperm‐mediated epigenetic inheritance [11, 12, 13]. Nevertheless, whether paternal preconception exposure can determine the maturation trajectory of the offspring's reproductive system via germ cell reprogramming remains unclear, with current knowledge lacking definitive causal evidence and detailed mechanistic insights.

The epigenetic landscape of paternal germ cells is widely recognized as a critical interface linking parental environmental exposures to offspring phenotypes [14, 15]. Among the various epigenetic mechanisms, DNA methylation at imprinting control regions (ICRs) plays a pivotal role by regulating parent‐of‐origin‐specific gene expression and can be partially preserved during genome‐wide epigenetic reprogramming following fertilization, thereby serving as an ideal carrier for intergenerational and transgenerational information transfer [16, 17]. Although alterations in imprinted loci in sperm have been associated with the multigenerational inheritance of metabolic traits [18, 19], the extent to which a given sperm methylation change is directly transmitted across post‐fertilization reprogramming and is sufficient to drive specific endocrine outcomes remains a major unresolved question in the field.

Caffeine, the most widely consumed central nervous system stimulant worldwide, has attracted significant scientific attention due to its high prevalence of consumption among individuals of reproductive age and its associated reproductive toxicity. Epidemiological evidence indicates that long‐term caffeine intake can impair sperm quality [20, 21] and induce a chronic stress state through activation of the hypothalamic‐pituitary‐adrenal (HPA) axis, resulting in persistently elevated serum glucocorticoid levels [22]. Our previous research has demonstrated that paternal preconception caffeine exposure (PPCE) can promote metabolic disorders in offspring via sperm‐mediated epigenetic mechanisms [19]. However, whether PPCE can drive autonomous ovarian steroidogenesis in offspring by reprogramming DNA methylation at key imprinted loci in sperm—and ultimately contribute to the development of PPP—remains to be elucidated.

This study aimed to determine whether PPCE influences ovarian endocrine programming and PPP‐like phenotypes in female offspring, and to define the sperm‐associated epigenetic mechanism involved. By integrating animal models, germ‐cell epigenetic analyses, IVF assays, ovarian gain‐ and loss‐of‐function experiments, GR antagonism, and exploratory human observations, we provide preclinical mechanistic evidence supporting a paternal glucocorticoid–associated sperm *Gnas*‐ICR mechanism that is linked to ovarian *Gnas* dysregulation and reproductive endocrine programming in offspring.

## 2 Results

### 2.1 PPCE Induces PPP‐Like Phenotypes and Autonomous Ovarian Steroidogenesis in Female Offspring Rats

**FIGURE 1:**
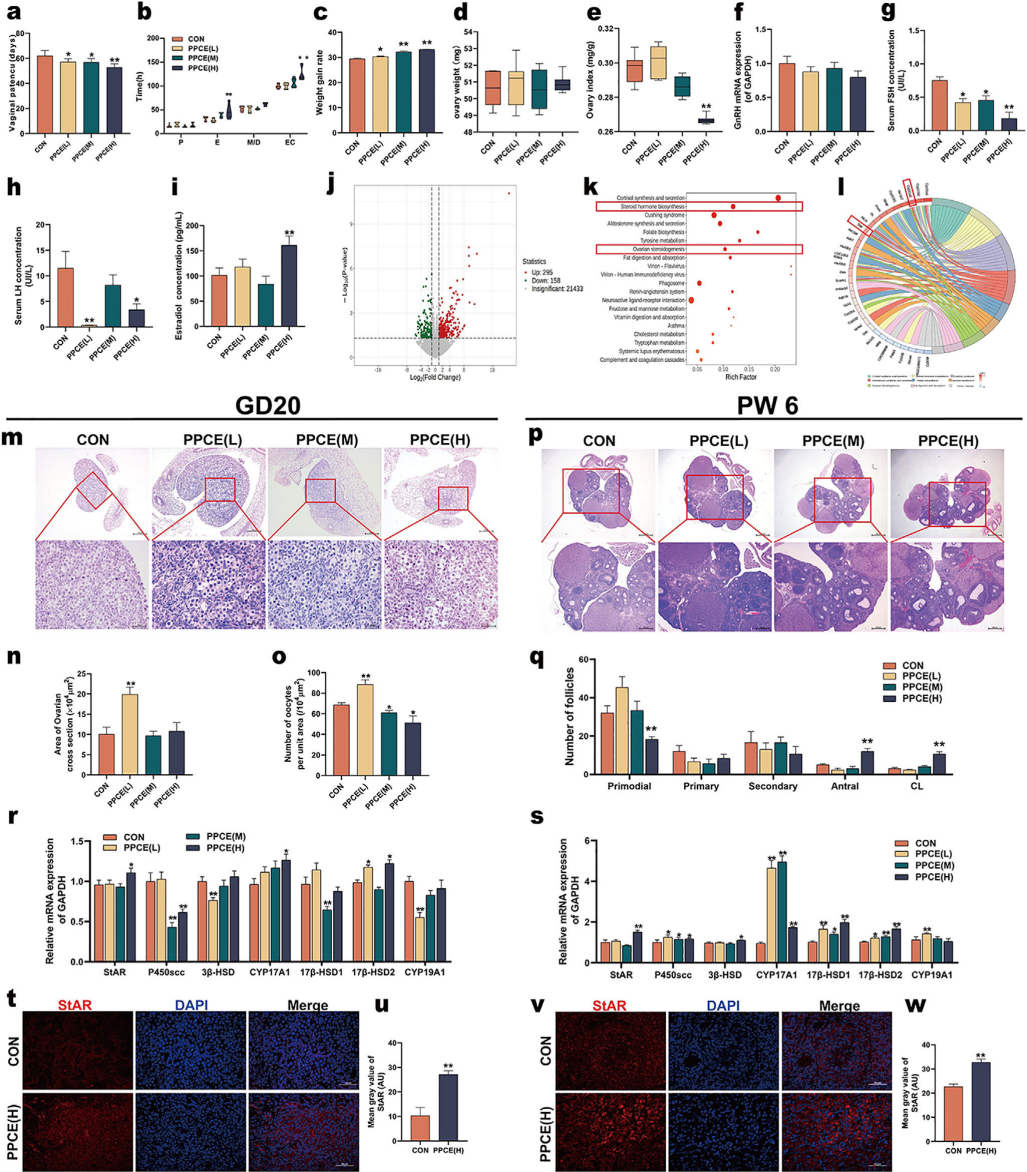
Effects of PPCE on PPP‐like phenotypes in female offspring rats. (a) Vaginal opening time, *n *= 12. (b) Estrous cycle, *n *= 12. (c) Weight gain rate at PW6, *n *= 12. (d) Ovarian weight at PW6, *n *= 12. (e) Ovarian index at PW6, *n *= 12. (f) Hypothalamic *GnRH* mRNA expression at PW6 and PW12 (Figure S1a), *n *= 12. (g–i) Serum FSH, LH, and E2 levels at PW6 and PW12 (Figure S1b–d), *n *= 12. (j) DEGs volcano map at GD20, *n *= 5. (k) KEGG functional enrichment analysis of DEGs at GD20. (l) DEGs at GD20. (m) Ovarian H&E staining at GD20 (100×, 400×). (n) Maximum ovarian cross‐sectional area at GD20, *n *= 5. (o) Oocyte number at GD20, *n *= 5. (p) Ovarian H&E staining at PW6 (20×, 40×). (q) Number of follicles at all developmental stages at PW6, *n *= 5. (r,s) Ovarian mRNA expression of *StAR, P450scc, 3β‐HSD1, CYP17A1, 17βHSD‐1, 17βHSD2* and *CYP19A1* at GD20, PW6 and PW12 (Figure S2c), *n *= 12. (t–w) StAR IF and quantification at GD20 and PW6 (400×, *n *= 5). Mean ± S.E.M. ^*^ *p* < 0.05, ^**^ *p *< 0.01 vs. CON group.

To clarify the impact of PPCE on the reproductive development of offspring, we systematically evaluated the pubertal phenotypes of female offspring. Our findings demonstrated that, compared with the CON group, the PPCE group exhibited a significantly earlier onset of vaginal opening, earlier initiation of estrus, and a prolonged estrous cycle. Additionally, the rate of weight gain was significantly increased, ovarian weight remained unchanged, but the ovarian index was notably reduced (Figure 1a–e). Endocrine analysis revealed that circulating estradiol (E2) levels in PPCE offspring were significantly elevated, whereas luteinizing hormone (LH) and follicle‐stimulating hormone (FSH) levels were decreased. This hormonal profile is consistent with “gonadotropin‐independent” estrogen excess rather than premature activation of the central hypothalamic‐pituitary‐gonadal (HPG) axis. Furthermore, hypothalamic gonadotropin‐releasing hormone (GnRH) expression was not upregulated (Figure 1f–i and Figure S1a–d), further excluding the possibility of central precocious puberty.

Transcriptome sequencing (RNA‐seq) analysis of GD20 fetal ovaries revealed that differentially expressed genes (DEGs) were predominantly enriched in the “steroid hormone biosynthesis” and “ovarian steroidogenesis” signaling pathways (Figures 1j–l). Morphological assessments confirmed that PPCE reduced the number of oocytes per unit area of the ovary prior to birth (Figures 1m–o), accompanied by a significant increase in antral follicles and corpora lutea postnatally at postnatal week 6 (PW6) (Figures 1p,q). The expression of key steroidogenic enzymes—including *StAR*, *P450scc*, and *CYP17A1*—was significantly upregulated across all developmental stages before and after birth (Figures 1r–w; Figure S2c). These findings collectively demonstrate that PPCE induces autonomous ovarian steroidogenesis in offspring, thereby contributing to PPP‐like phenotypes.

### 2.2 PPCE‐Associated Sperm *Gnas*‐ICR Hypermethylation Is Linked to Ovarian Gnas Upregulation and PPP‐Like Phenotypes in Offspring

**FIGURE 2:**
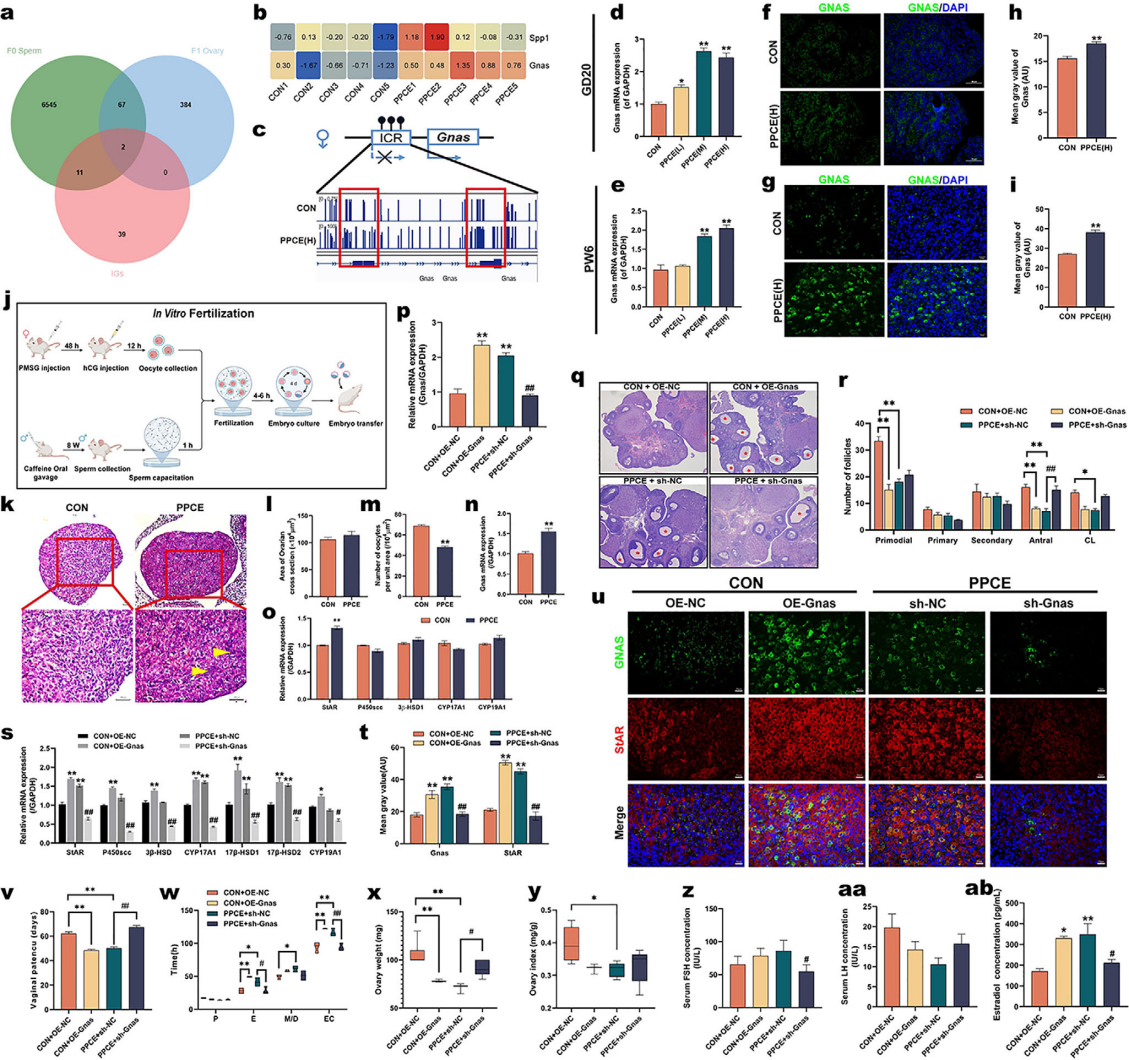
Functional contribution of ovarian Gnas dysregulation to PPCE‐induced PPP‐like phenotypes in female offspring rats. (a) Venn diagram intergrating parental sperm methylation‐alerted genes, offspring ovarian DEGs and imprinted genes. (b) GD20 ovarian imprinted gene expression, *n *= 5. (c) *Gnas*‐ICR methylation in F0 sperm. (d,e) *Gnas* mRNA expression at GD20 and PW6, *n *= 12. (f‐i) GNAS immunofluorescence and protein expression at GD20 and PW6, *n *= 5. (j) Experimental design of the mouse IVF assay. (k) PPCE IVF offspring ovaries H&E staining at GD19 (100×, 400×). (l) Maximum ovary cross‐sectional area, *n *= 5. (m) Oocyte number per unit area, *n* = 5. (n) *Gnas* mRNA expression in GD19 ovaries derived from PPCE‐IVF offspring, *n *= 12. (o) mRNA expression of *StAR, P450scc, 3β‐HSD1, CYP17A1*, and *CYP19A1* in GD19 ovaries derived from PPCE‐IVF offspring, *n *= 12. (p) *Gnas* mRNA expression at PW12, *n *= 10. (q) H&E staining at PW12 (20×, 40×), *n *= 5 (*shows cystic follicle). (r) Follicle counts at PW12, *n *= 5. (s,t) GNAS and StAR IF and quantification at PW12, *n *= 5. (u) Estrogen synthesis‐related genes mRNA expression at PW12, *n *= 10. v, Vaginal opening time, *n *= 10. w, Estrus cycle, *n *= 10. x, Ovarian weight at PW12, *n *= 10. (y) Ovarian index at PW12, *n *= 10. (z‐ab) Serum FSH, LH and E2 levels at PW12, *n *= 10. Mean ± S.E.M. ^*^ *p *< 0.05, ^**^ *p* < 0.01 vs. CON group.

To identify sperm‐associated epigenetic candidates potentially linked to enhanced ovarian steroidogenesis in offspring induced by PPCE, we first performed an integrated analysis of whole‐genome bisulfite sequencing (WGBS) data from parental sperm, RNA‐seq data from offspring ovaries, and the imprinted gene dataset (Geneimprint:Genes). The results revealed that two imprinted genes were altered in both paternal sperm (based on DNA methylation profiles) and the ovaries of the offspring (based on transcriptomic profiles) (Figure 2a). Among these candidates, Gnas showed the most prominent change, with increased methylation at the *Gnas* imprinting control region (ICR) in paternal sperm and increased expression in offspring ovaries (Figure 2b,c). We next validated ovarian Gnas expression in offspring. Both mRNA and protein levels of Gnas were significantly increased in PPCE offspring ovaries at GD20 and PW6 (Figure 2d–i). To further examine whether *Gnas*‐ICR hypermethylation was associated with altered transcriptional regulation within the *Gnas* imprinted domain, we measured the expression of *Nespas*, an antisense transcript located in this locus. PPCE significantly reduced ovarian *Nespas* expression in female offspring, accompanied by increased *Gnas* expression at the same developmental stage (Figure S3a). We further analyzed *Gnas*‐ICR methylation in F1 ovaries at GD20, PW6, and PW12. PPCE‐associated *Gnas*‐ICR hypermethylation was observed across these examined prenatal and postnatal ovarian stages (Figure S3d–i). These data indicate an association between ovarian *Gnas*‐ICR hypermethylation, reduced Nespas expression, and increased Gnas expression during the examined developmental stages. However, because zygote/ZGA‐stage methylation and targeted manipulation of the *Gnas*‐ICR/Nespas module were not directly assessed, these findings do not establish either a direct causal sequence among *Gnas*‐ICR methylation, Nespas repression, and Gnas activation, or uninterrupted maintenance of this methylation mark from fertilization through early embryogenesis.

To determine whether sperm‐associated information from PPCE males was sufficient to influence offspring ovarian development, we performed a mouse IVF assay using sperm from CON or PPCE males and oocytes from untreated females (Figure 2j). Compared with the CON‐IVF group, fetal ovaries derived from PPCE sperm displayed abnormal ovarian morphology and development, including reduced ovarian size and decreased oocyte number per unit area (Figure 2k–m). In parallel, Gnas expression and the mRNA expression of steroidogenic genes, including *StAR, P450scc*, *3β‐HSD1, CYP17A1*, and *CYP19A1*, were increased in fetal ovaries derived from PPCE sperm (Figure 2n,o). These findings indicate that sperm‐associated information from PPCE males can contribute to early ovarian developmental abnormalities and increased Gnas‐related steroidogenic signaling in offspring.

Next, we performed AAV9‐mediated ovarian *Gnas* gain‐ and loss‐of‐function experiments in female offspring. In CON offspring, ovarian Gnas overexpression partially recapitulated PPCE‐like ovarian pathological changes, enhanced GNAS/StAR co‐localization, increased the expression of estrogen synthesis‐related genes, and induced PPP‐like phenotypes, including earlier vaginal opening, disrupted estrous cyclicity, reduced ovarian index, increased serum E2 levels, and decreased LH levels (Figure 2p–ab). Conversely, Gnas knockdown in PPCE offspring significantly ameliorated ovarian pathological changes, reduced steroidogenic gene expression, and improved PPP‐like phenotypes and endocrine alterations (Figure 2p–ab). These gain‐ and loss‐of‐function data indicate that ovarian Gnas dysregulation functionally contributes to PPCE‐induced enhancement of ovarian steroidogenesis and PPP‐like phenotypes in female offspring.

### 2.3 Gnas Overexpression Promotes Ovarian Steroid Synthesis Through cAMP/PKA/CREB Signaling

**FIGURE 3:**
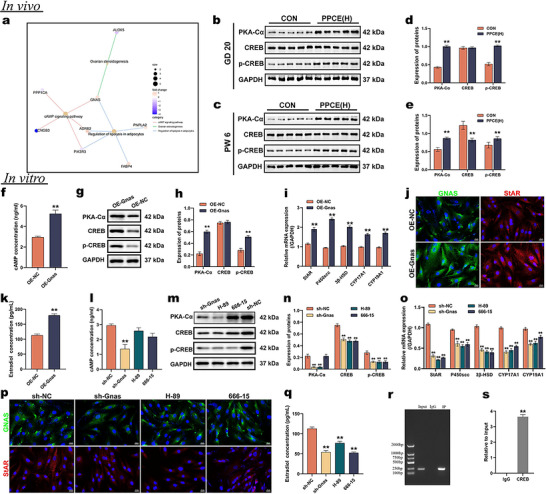
Gnas promotes ovarian steroid synthesis through the cAMP/PKA/CREB signaling pathway. (a) Interaction between Gnas and cAMP signaling pathway. (b,d) cAMP signaling pathway‐related protein expression at GD20, *n *= 5. (c,e) cAMP signaling pathway‐related protein expression at PW6, *n *= 5. (f) *Gnas* overexpression intracellular cAMP concentration, *n *= 6. (g,h) *Gnas* overexpression PKA‐Cα, CREB, and p‐CREB protein expression, *n *= 3. (i) *Gnas* overexpression estrogen synthesis‐related genes mRNA expression, *n *= 6. (j) GNAS and StAR IF images, *n *= 3. (k) *Gnas* overexpression culture medium E2 concentration, *n *= 6. (l) GNAS/PKA/CREB inhibition‐treated cell culture medium cAMP concentration, *n *= 6. (m,n) GNAS/PKA/CREB inhibition treated cell PKA‐Cα, CREB, and p‐CREB protein expression, *n *= 3. (o) GNAS/PKA/CREB inhibition treated cell estrogen synthesis‐related genes mRNA expression, *n *= 6. (p) GNAS/PKA/CREB inhibition treated cell GNAS and StAR IF images, *n *= 3. (q) GNAS/PKA/CREB inhibition treated cell culture medium E2 concentration, *n *= 6. (r,s) CREB binding to the StAR promoter, *n *= 3. Mean ± S.E.M. ^*^ *p *< 0.05, ^**^ *p *< 0.01 vs. CON group.

We further explored the molecular mechanism through which high expression of Gnas mediates enhanced estrogen synthesis in the ovaries of female offspring rats in the PPCE group. First, the RNA‐seq data indicated an interaction among ovarian steroidogenesis, the cAMP signaling pathway, and Gnas (Figure 3a). Subsequently, the expression of key proteins in the cAMP signaling pathway, PKA‐Cα and p‐CREB, in the ovaries of the PPCE group at GD20 and PW6 was significantly higher than those in the CON group (Figure 3b–e). Furthermore, we transfected OE‐*Gnas* or sh‐*Gnas* plasmids into the human ovarian granulosa cell line KGN to overexpress or knock down Gnas. Results showed that compared with the OE‐NC group, Gnas overexpression significantly increased cAMP concentration, the expression of PKA‐Cα and p‐CREB proteins (Figure 3f–h), mRNA expression of estrogen synthesis‐related enzyme genes, and StAR protein expression, as well as E2 concentration (Figure 3i–k). In contrast, Gnas knockdown significantly reversed these effects. To test pathway dependence, we treated cells with the PKA inhibitor H‐89 or the CREB inhibitor 666‐15. cAMP concentration remained unchanged, whereas p‐CREB protein levels were significantly reduced, and the mRNA expression of estrogen synthesis enzymes, StAR protein expression, and E2 concentration all decreased significantly (Figure 3l–q). Chromatin immunoprecipitation (ChIP)‐qPCR experiments further confirmed the binding of CREB to the StAR promoter region (Figure 3r,s). In conclusion, the activation of the cAMP/PKA/CREB signaling pathway mediates enhanced ovarian steroidogenic function in female offspring rats with elevated Gnas expression.

### 2.4 Paternal Glucocorticoid/GR Signaling Contributes to PPCE‐associated Sperm *Gnas*‐ICR Methylation Remodeling and Offspring PPP‐Like Phenotypes

**FIGURE 4:**
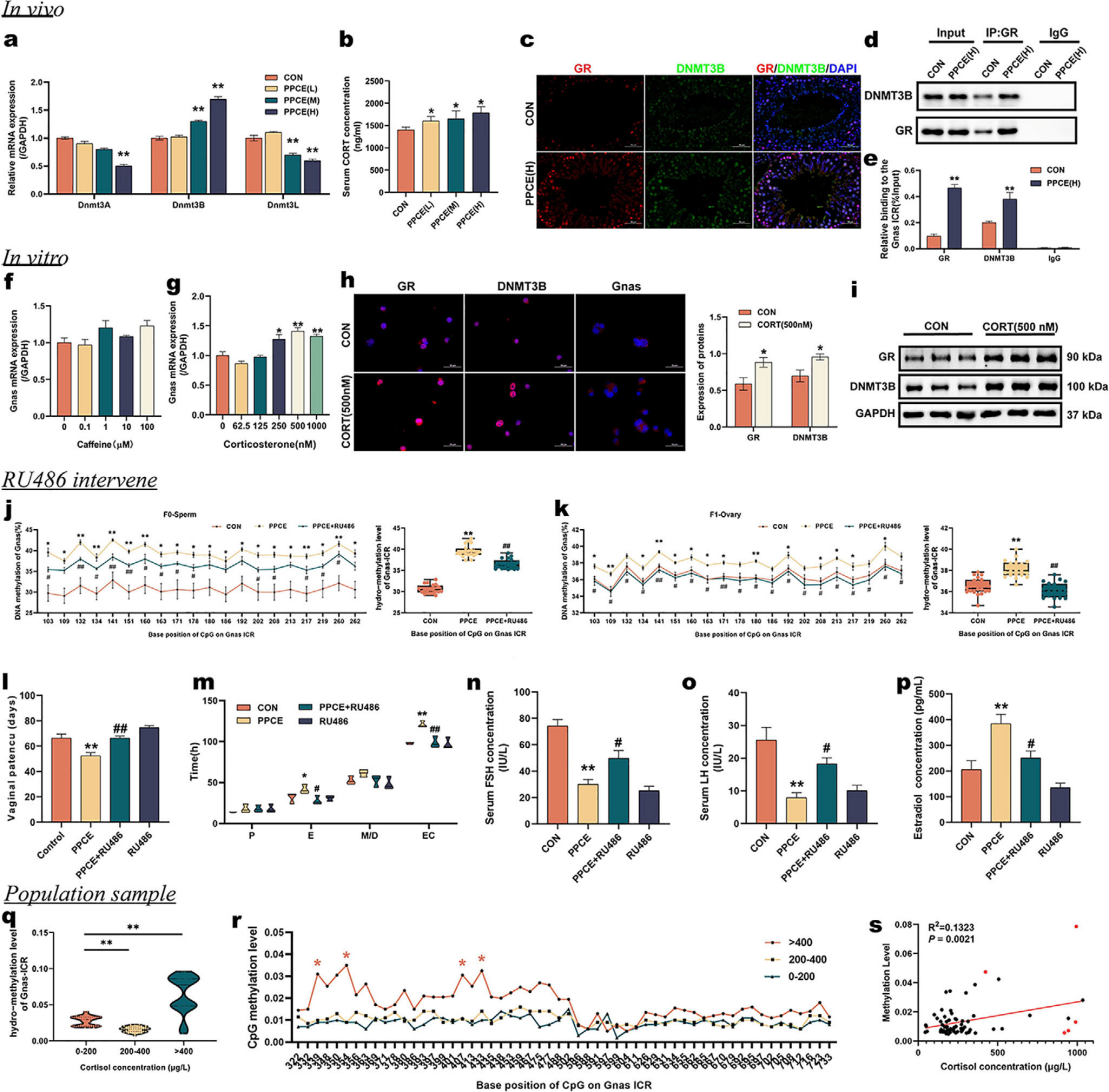
Glucocorticoid/GR‐dependent programming of sperm *Gnas*‐ICR methylation and RU486 rescue of offspring PPP‐like phenotypes. (a) Testis *Dnmt3A*, *Dnmt3B* and *Dnmt3L* mRNA expression, *n *= 12. (b) Serum corticosterone content, *n *= 12. (c) Testicular GR and DNMT3B IF images, *n *= 5. (d) Testicular GR and DNMT3B protein interaction. (e) ChIP‐qPCR analysis showing GR and DNMT3B enrichment at the *Gnas*‐ICR in paternal testis tissues. (f,g) *Gnas* mRNA expression of caffeine and corticosterone‐treated spermatogonia, *n *= 6. (h) GR and DNMT3B IF images of corticosterone‐treated spermatogonia, *n *= 3. (i) Corticosterone‐treated spermatogonia GR and DNMT3B protein expression, *n *= 3. (j,k) *Gnas*‐ICR methylation levels of F0 sperm and F1‐GD20 ovaries, *n *= 3. (l) Vaginal opening time, *n *= 12. (m) Estrous cycle, *n *= 12. (n–p) Serum FSH, LH, and E2 levels, *n *= 12. (q) Sperm *Gnas*‐ICR methylation levels in reproductive‐aged men stratified by plasma cortisol concentration. (r) CpG‐site‐specific methylation levels within the sperm *Gnas*‐ICR across cortisol‐stratified groups. (s) Scatter plot showing the exploratory association between plasma cortisol concentration and sperm *Gnas*‐ICR methylation in reproductive‐aged men with valid paired measurements (*n* = 69). The five influential observations identified by Cook's distance are highlighted in red and were retained in this primary plot to display the true data distribution. Mean ± S.E.M. For animal experiments, ^*^ *p *< 0.05, ^**^ *p *< 0.01 vs. CON group. For human analyses, statistical details are provided in Tables S1 and S2.

We further investigated a candidate upstream process associated with PPCE‐induced hypermethylation of the *Gnas*‐ICR in paternal sperm. First, we examined the expression levels of a panel of DNA methyltransferases in the testes of the F0 generation, revealing that DNA methyltransferase 3B (*Dnmt3b*) mRNA expression was significantly upregulated in the PPCE group (Figure 4a). Concurrently, PPCE induced a sustained glucocorticoid‐elevated state in fathers, as evidenced by elevated serum corticosterone levels (Figure 4b). In paternal testes, both protein expression and physical interaction between the glucocorticoid receptor (GR) and DNMT3B were enhanced (Figure 4c,d). To further determine whether these factors were recruited to the imprinted locus, we performed chromatin immunoprecipitation coupled with qPCR (ChIP‐qPCR) on paternal testis tissues. The results revealed significantly enhanced enrichment of both GR and DNMT3B at the *Gnas*‐ICR region in the PPCE group (Figure 4e). In vitro experiments using spermatogonia showed that, compared with the CON group, caffeine treatment at various concentrations did not significantly alter *Gnas* expression (Figure 4f), whereas exposure to high concentrations of corticosterone (500 nM) significantly increased *Gnas* mRNA levels, along with upregulated protein expression of GR and DNMT3B (Figure 4g–i). Together, these findings support a model in which elevated paternal glucocorticoid signaling promotes GR/DNMT3B recruitment to the *Gnas*‐ICR and contributes to locus‐associated methylation remodeling.

Finally, at the whole‐animal level, administration of the GR antagonist RU486 attenuated PPCE‐induced hypermethylation of the *Gnas*‐ICR in F0 sperm and F1 ovaries and ameliorated PPP‐like phenotypes in F1 female offspring, including earlier vaginal opening, prolonged estrous cycles, reduced circulating LH levels, and elevated E2 levels (Figure 4j–p and Figure S4). Paternal RU486 intervention also partially restored the reduced ovarian *Nespas* expression in PPCE offspring, consistent with attenuation of *Gnas*‐ICR hypermethylation and ovarian Gnas dysregulation (Figure S4e). These findings support the interpretation that elevated paternal glucocorticoid levels participate in the epigenetic programming of sperm *Gnas*‐ICR methylation following PPCE and contribute to PPP‐like phenotypes in female offspring.

To explore the potential human relevance of the glucocorticoid‐associated sperm *Gnas*‐ICR methylation pattern observed in the animal model, we analyzed clinical samples from 77 reproductive‐aged men. Among them, 69 subjects had valid paired measurements of plasma cortisol concentration and sperm *Gnas*‐ICR methylation and were included in the primary analysis. Men with higher plasma cortisol levels (>400 µg/L) showed increased overall and CpG‐site‐specific sperm *Gnas*‐ICR methylation compared with those with lower cortisol levels (Figure 4q,r). In the primary univariate Pearson analysis including all 69 valid paired samples, plasma cortisol concentration was positively associated with sperm *Gnas*‐ICR methylation (R^2^ = 0.1323, *p *= 0.0021; Figure 4s). However, this association was influenced by several observations identified by Cook's distance and was not consistently supported by all sensitivity analyses, including ROUT‐based outlier analysis, Spearman correlation, and cortisol‐stratified regression analyses (Table S1).

In an exploratory adjusted model including available covariates, plasma cortisol concentration remained associated with sperm *Gnas*‐ICR methylation (*p* = 0.0010; model R^2^ = 0.1899; Table S2). Nevertheless, because precise caffeine intake, alcohol consumption, validated psychological stress scores, and longitudinal offspring outcomes were unavailable, residual confounding cannot be excluded. These human data should therefore be interpreted as exploratory and hypothesis‐supporting evidence consistent with the animal mechanism rather than as clinical validation of a conserved causal pathway.

### 2.5 Paternal‐Line Persistence of PPCE‐Associated PPP‐Like Phenotypes and Ovarian Endocrine Programming in F2 Offspring

**FIGURE 5:**
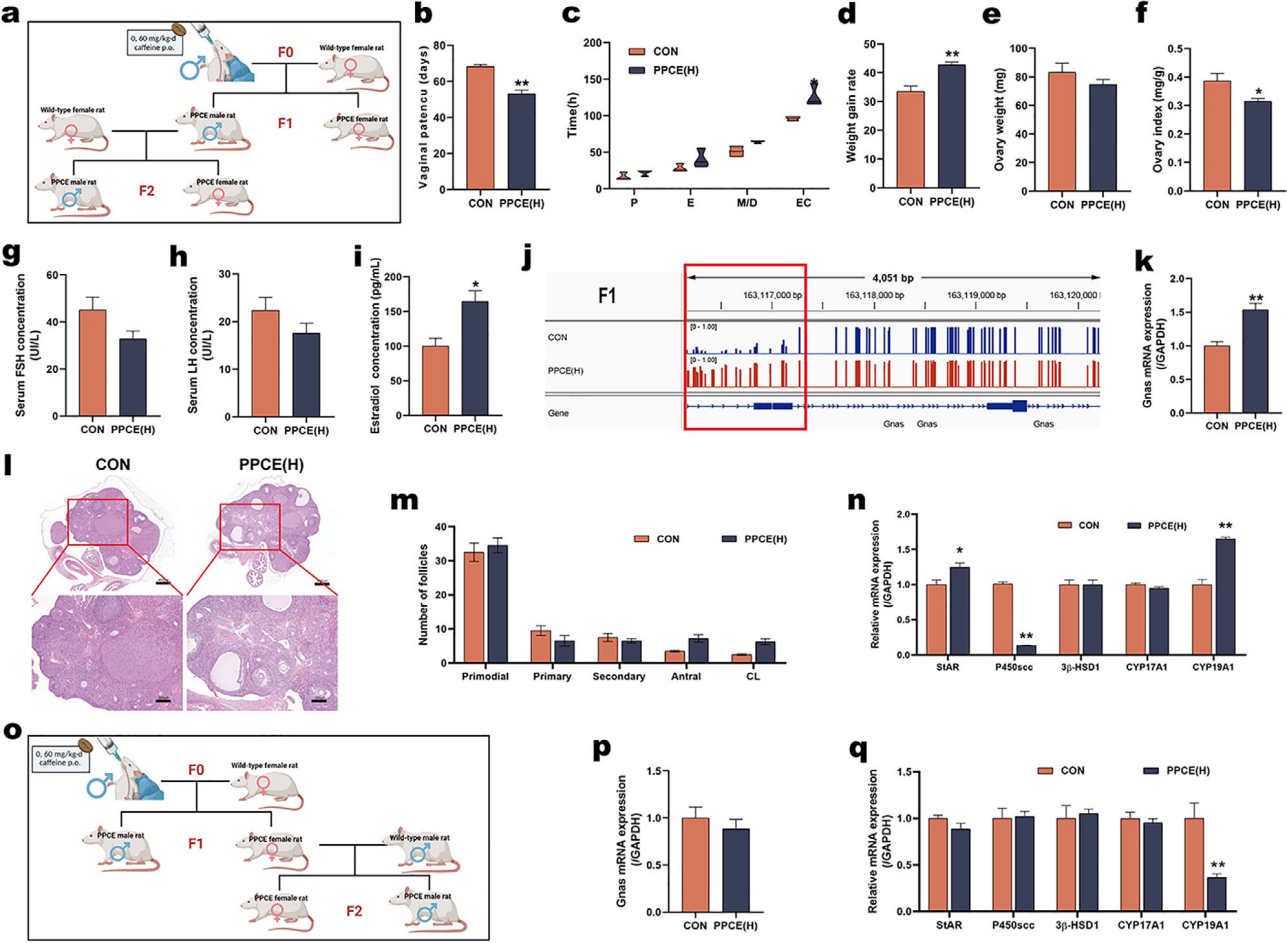
Paternal‐line persistence of PPCE‐associated PPP‐like phenotypes and ovarian endocrine programming in F2 offspring. (a) Paternal F2 generation diagram. (b) F2 vaginal opening time, *n *= 12. (c) F2 estrous cycle, *n *= 12. (d) F2 weight gain at PW6, *n *= 12. (e) F2 ovarian weight at PW6, *n *= 12. (f) F2 ovarian index at PW6, *n *= 12. (g–i) F2 serum FSH, LH and E2 levels at PW6, *n *= 12. (j) F1 sperm *Gnas*‐ICR methylation level at PW16. (k) F2 *Gnas* mRNA expression at PW6, *n *= 12. (l) Representative F2 ovarian H&E staining at PW6 (20× and 40×). Corresponding PW12 H&E staining is shown in Figure S5d. (m) F2 follicle counts at PW6, *n *= 5. Corresponding PW12 follicle counts are shown in Figure S5e. (n) F2 *StAR, P450scc, 3β‐HSD1, CYP17A1*, and *CYP19A1* mRNA expression at PW6 and PW12 (Figure S5g), *n *= 12. (o) Maternal line F2 generation diagram. (p) Ovarian *Gnas* mRNA expression of maternal line F2 generation at PW12, *n *= 12. (q) *StAR, P450scc, 3β‐HSD1, CYP17A1* and *CYP19A1* mRNA expression of maternal line F2 generation at PW12, *n *= 12. Mean ± S.E.M. ^*^ *p *< 0.05, ^**^ *p *< 0.01 vs. CON group.

In the model used in this study, the effects observed in the F1 generation are best interpreted as intergenerational effects, because F1 offspring were directly derived from sperm exposed to the paternal preconception environment [23, 24]. In contrast, the PPP phenotypes observed in the F2 generation reflect paternal‐line cross‐generational persistence. To evaluate the persistence of PPCE‐induced effects across generations, we further monitored the phenotypic manifestations in the paternal F2 generation (Figure 5a). The results demonstrated that, compared to the F2‐CON group, the F2‐PPCE group exhibited earlier vaginal opening, prolonged estrous cycles, a higher body weight gain rate at PW6, a reduced ovarian index, decreased serum FSH and LH levels, and elevated serum E2 levels (Figure 5b–i). Together, these phenotypic and molecular alterations indicate that PPCE‐associated ovarian endocrine changes persisted in paternal‐line F2 offspring. Further results revealed that the methylation level of *Gnas*‐ICR in F1‐PPCE sperm and *Gnas* mRNA expression in F2‐PPCE ovaries (Figure 5j,k) were significantly increased. Meanwhile, there was no significant difference in the number of follicles, but the mRNA expression of steroidogenic enzymes, including *StAR* and *CYP19A1*, was significantly elevated in the ovaries of the PPCE group at PW6 (Figure 5l–n). Similar endocrine, histological, and molecular findings were observed in the PPCE group at PW12 (Figure S5a–g). In contrast, maternal‐line F2 offspring did not show comparable ovarian Gnas upregulation or steroidogenic gene activation (Figure 5o–q), suggesting that the PPCE‐associated ovarian molecular effect was preferentially transmitted through the paternal line under the present experimental conditions. These findings suggest paternal‐line persistence of PPCE‐associated ovarian endocrine programming, whereas maternal‐line transmission was not evident under the present experimental conditions.

## 3 Discussion

This study provides preclinical evidence that paternal preconception caffeine‐associated glucocorticoid elevation can influence offspring ovarian endocrine development through sperm‐associated epigenetic programming. Building on emerging evidence that paternal environmental exposures can influence offspring phenotypes through sperm‐borne epigenetic information [11, 14, 15, 16], our findings further extend this framework to paternal preconception endocrine disturbance and offspring reproductive developmental programming. Using a PPCE rat model, IVF, ovarian *Gnas* gain‐ and loss‐of‐function, GR antagonism, and exploratory human samples, we provide evidence supporting a paternal glucocorticoid‐associated sperm *Gnas*‐ICR mechanism linked to ovarian Gnas dysregulation and PPP‐like phenotypes in female offspring.

The PPCE protocol was designed to establish a biologically interpretable model of paternal preconception exposure rather than to define clinically equivalent caffeine doses or human risk thresholds. Population‐based data indicate that men of reproductive age consume an average of approximately 240 mg of caffeine per day, with high consumers possibly reaching 828 mg/d [25, 26]. Based on the surface area conversion between humans and rats (human:rat = 1:6.17) [27], these values roughly correspond to 21.2 mg/kg.d and 73 mg/kg.d in rats. Therefore, the doses selected for this study (15, 30, and 60 mg/kg/d) were intended to cover a biologically relevant exposure range for mechanistic modeling. The highest dose is broadly comparable to the caffeine intake that may be achieved by several cups of brewed coffee per day, depending on serving size and preparation method [28]. The 8‐week exposure period was chosen to cover the full spermatogenic cycle in rats, which lasts approximately 52–54 days [29]. Thus, this protocol provides an experimental model for investigating PPCE‐associated paternal sperm reprogramming, offspring ovarian endocrine programming, and potential mechanistic intervention targets, but it should not be interpreted as defining a clinical risk threshold for human caffeine intake.

Mechanistically, our data support glucocorticoid signaling as a candidate upstream regulator of PPCE‐associated sperm *Gnas*‐ICR methylation. This interpretation is consistent with previous evidence that glucocorticoid/GR signaling can influence DNMT expression and activity [30]. In our model, PPCE induced a sustained paternal glucocorticoid‐elevated state, and corticosterone, but not caffeine, activated GR/DNMT3B‐related responses in spermatogonial cells. Given the established role of DNMT3‐family enzymes in de novo methylation at germline and imprinted regions [31, 32], we further examined whether GR and DNMT3B were associated with the *Gnas*‐ICR. Co‐IP and ChIP‐qPCR supported the interaction between GR and DNMT3B and their enrichment at the *Gnas*‐ICR region, while paternal RU486 intervention attenuated sperm *Gnas*‐ICR hypermethylation and offspring PPP‐like phenotypes. Together, these findings support a model in which paternal glucocorticoid/GR signaling is linked to GR/DNMT3B‐associated methylation remodeling at the *Gnas* imprinted domain, although they do not prove a direct linear cascade.

The Gnas locus is a complex imprinted region that contains multiple protein‐coding and non‐coding transcripts with parent‐of‐origin specificity and tissue specificity [33]. Previous genetic studies have shown that the Nespas‐associated differentially methylated region functions as a major imprinting control region within the Gnas cluster and can influence the expression of multiple transcripts in this locus, including Gnas [34, 35]. In addition, Nespas is a paternally expressed antisense macroRNA arising from this regulatory region, and experimental perturbation of Nespas transcription has been shown to affect local chromatin state, methylation, and transcript regulation within the Gnas imprinted domain [36, 37]. These findings provide a biologically plausible framework in which altered methylation at the *Gnas*‐ICR may be linked to changes in Nespas expression and downstream Gnas regulation. In this study, PPCE‐associated *Gnas*‐ICR hypermethylation was detected in F0 sperm and observed in F1 offspring ovaries at GD20, PW6, and PW12. This pattern was accompanied by decreased Nespas expression and increased Gnas expression in offspring ovaries. In addition, paternal RU486 intervention restored or partially restored Nespas expression in the ovaries, while reducing *Gnas*‐ICR hypermethylation and PPP‐like phenotypes, further supporting the involvement of paternal glucocorticoid/GR signaling in this regulatory process. Thus, our data are consistent with a literature‐supported regulatory relationship among *Gnas*‐ICR methylation, Nespas repression, and Gnas upregulation in PPCE‐associated ovarian endocrine programming. However, because targeted manipulation of *Gnas*‐ICR methylation or Nespas was not performed in the present study, these findings should be interpreted as an association‐supported and biologically plausible regulatory link rather than proof of a direct causal *Gnas*‐ICR/Nespas/Gnas pathway.

At the effector level, GNAS encodes the stimulatory G protein α‐subunit (Gsα), a central node in cAMP‐dependent signal transduction across endocrine tissues [38, 39]. In ovarian cells, activation of the cAMP/PKA/CREB pathway promotes the transcription of steroidogenic regulators, including StAR, thereby enhancing estrogen biosynthesis [40]. In this study, increased ovarian Gnas expression in PPCE offspring was accompanied by enhanced cAMP/PKA/CREB signaling. In granulosa cells, Gnas overexpression increased cAMP accumulation, PKA‐Cα and p‐CREB activation, StAR expression, and E2 production, whereas Gnas knockdown or inhibition of PKA/CREB attenuated these responses. Consistently, ovarian Gnas overexpression partially recapitulated PPCE‐like steroidogenic and pubertal phenotypes in control offspring, while Gnas knockdown attenuated these alterations in PPCE offspring. These findings support the GNAS–cAMP–PKA–CREB–StAR pathway as a downstream effector mechanism through which ovarian Gnas dysregulation contributes to PPCE‐induced ovarian endocrine programming.

Using IVF with sperm from PPCE‐exposed males and oocytes from unexposed females, we showed that sperm‐associated information from PPCE males was capable of influencing early ovarian developmental abnormalities and steroidogenic gene upregulation in F1 offspring. In the paternal‐line F2 generation, offspring also showed PPP‐like phenotypes, increased ovarian Gnas expression, and enhanced steroidogenic gene expression, whereas maternal‐line F2 offspring did not show comparable ovarian endocrine alterations. These findings indicate paternal‐line transmission of PPCE‐associated phenotypes, but do not provide sufficient evidence to establish *Gnas*‐ICR methylation as a heritable epigenetic determinant of disease. Notably, the F2 phenotype was milder than that observed in F1 offspring, suggesting that this inherited effect is developmentally plastic rather than permanently fixed. This interpretation is consistent with the concept that epigenetic inheritance is subject to germline and post‐fertilization remodeling, which can modify the persistence and magnitude of transmitted phenotypes [16]. Thus, the F2 data support paternal‐line persistence of PPCE‐associated ovarian endocrine programming, but do not by themselves prove uninterrupted maintenance of a specific *Gnas*‐ICR methylation mark across fertilization, ZGA, and subsequent generations.

The exploratory human data provide a preliminary translational link. Plasma cortisol was associated with sperm *Gnas*‐ICR methylation in the primary and adjusted models, but the association was weak, method‐sensitive, and not consistently supported across all sensitivity analyses. Moreover, the cohort lacked precise caffeine intake data, validated psychological stress measures, alcohol consumption information, and longitudinal offspring pubertal outcomes. Therefore, these human findings should be viewed as hypothesis‐generating evidence consistent with the animal mechanism, not as clinical validation. Future prospective studies integrating paternal caffeine intake, stress assessment, cortisol dynamics, sperm epigenetic markers, and offspring pubertal outcomes will be required to test clinical relevance.

The magnitude of PPCE‐associated sperm *Gnas*‐ICR methylation change was modest in bulk sperm DNA analysis. This should be interpreted cautiously, because bulk methylation measurements represent an average across heterogeneous sperm populations and may underestimate CpG‐site‐specific or subpopulation‐specific changes [41, 42]. ICRs are key regulatory elements for parent‐of‐origin‐specific gene expression, and their functional impact may depend on locus context, CpG position, allele specificity, and interaction with downstream transcriptional regulation [17]. Because ICRs function as allele‐specific regulatory elements, even relatively small locus‐specific methylation shifts may be biologically relevant if they occur at functionally important CpG sites or within specific sperm subpopulations [35, 43]. Nevertheless, the present data do not demonstrate that the observed methylation change alone is sufficient to account for all downstream phenotypes. Other sperm‐borne epigenetic information and postfertilization developmental remodeling may also contribute to the observed transcriptional and phenotypic outcomes [14, 16]. Therefore, *Gnas*‐ICR methylation remodeling should be viewed as one component of a broader paternal glucocorticoid‐associated sperm and ovarian regulatory network.

Several limitations should be acknowledged. First, allele‐specific expression and allele‐specific methylation analyses were not feasible in the current Wistar rat model, which was not designed with genetically informative reciprocal crosses. Second, although *Gnas*‐ICR hypermethylation was observed from F0 sperm to F1 ovaries across GD20, PW6, and PW12, methylation dynamics during fertilization and ZGA remain to be directly examined. Third, the magnitude of sperm *Gnas*‐ICR methylation increase was modest; although imprinting control regions can be sensitive to methylation perturbation, the current data do not determine whether this degree of methylation change is by itself sufficient to account for all downstream transcriptional and phenotypic alterations. Accordingly, the observed methylation difference should be interpreted as one component of a broader sperm‐associated and ovarian regulatory network. Fourth, the human analysis was exploratory, limited in sample size, and lacked complete information on caffeine intake, alcohol consumption, psychological stress, and longitudinal offspring outcomes. Finally, PPP‐like phenotypes likely reflect a multifactorial regulatory network, and our ongoing observations suggest that maternal regulatory influences may also affect ovarian imprinted‐gene programming and postnatal phenotypic attenuation.

## 4 Conclusions

**FIGURE 6:**
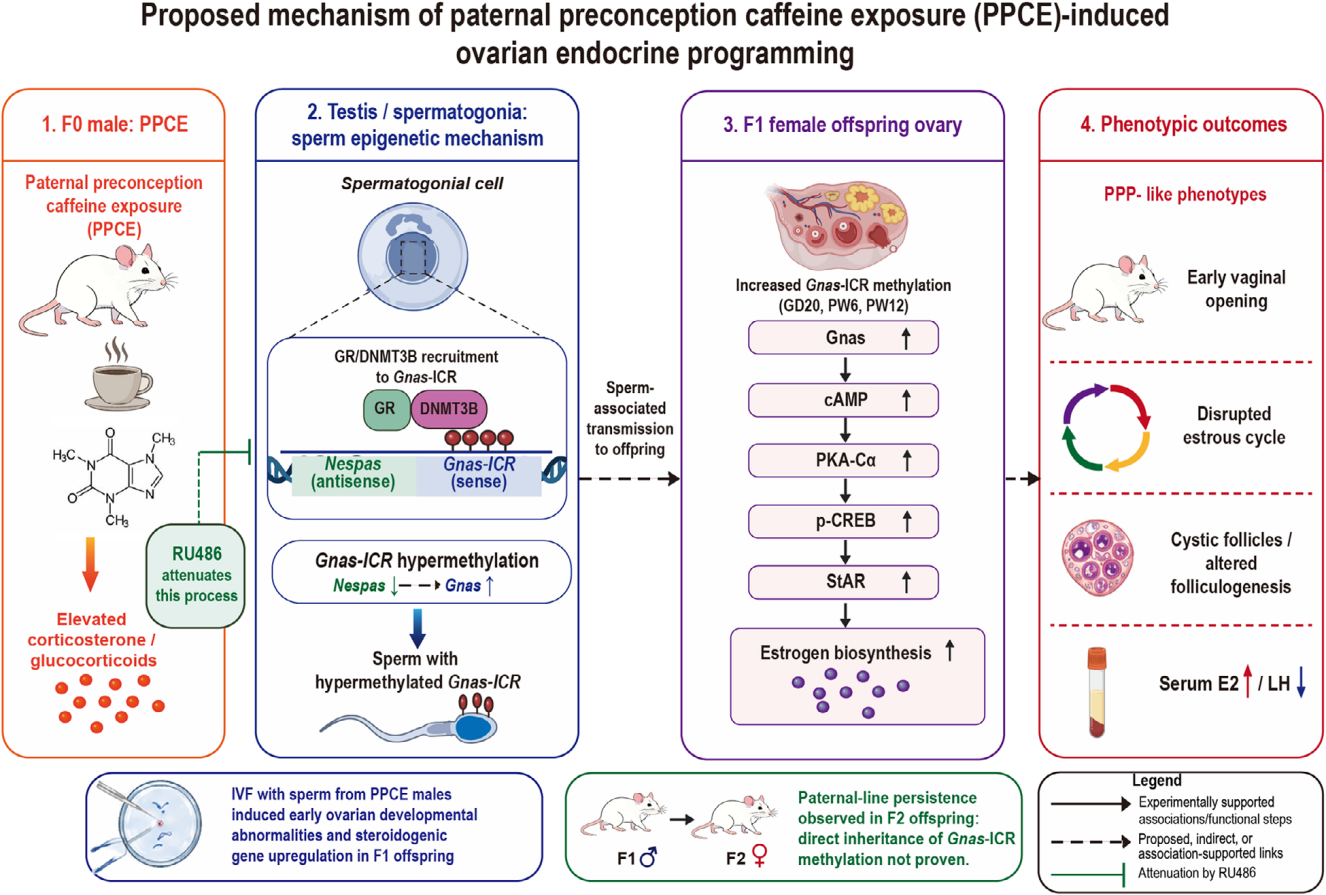
Proposed model of paternal glucocorticoid–associated sperm *Gnas*‐ICR epigenetic programming in PPCE‐associated offspring ovarian endocrine dysregulation and PPP‐like phenotypes. Solid arrows indicate experimentally supported associations or functional steps in the present study, whereas dashed arrows indicate proposed or indirect links that require further validation.

This study provides preclinical evidence that paternal preconception caffeine‐associated glucocorticoid elevation can influence offspring ovarian endocrine programming in association with sperm *Gnas*‐ICR epigenetic remodeling. Mechanistically, this process is associated with GR/DNMT3B recruitment to the sperm *Gnas*‐ICR, altered ovarian Nespas/Gnas expression, and activation of the cAMP/PKA/CREB pathway, leading to enhanced ovarian steroidogenesis and PPP‐like phenotypes. The detection of *Gnas*‐ICR hypermethylation in F0 sperm and in F1 ovaries at GD20, PW6, and PW12 supports developmental persistence during the examined prenatal and postnatal ovarian stages, while paternal‐line F2 findings suggest cross‐generational persistence of PPCE‐associated ovarian endocrine programming (Figure 6). These findings expand the mechanistic framework of paternal DOHaD in reproductive endocrine development. Although direct zygote/ZGA‐stage methylation dynamics and clinical relevance remain to be established, the present study provides a mechanistic basis for future investigations into how paternal preconception endocrine status may influence offspring reproductive development.

## 5 Materials and Methods

### 5.1 Reagents and Cell Culture

#### 5.1.1 Reagents

Caffeine (purity ≥ 99.0%) and corticosterone were purchased from Sigma‐Aldrich (St Louis, MO, USA). Mifepristone (RU486) was obtained from Hubei Gedian Humanwell Pharmaceutical Co., Ltd. (Ezhou, China). Plasmids (Hm‐sh‐*Gnas*, Hm‐OE‐*Gnas*) and adeno‐associated viruses (AAV9‐*Gnas*, sh‐*Gnas*) were synthesized by Yunzhou Biotechnology (Guangzhou, China); their sequences are shown in Table S3. Follicle‐stimulating hormone (FSH) and luteinizing hormone (LH) ELISA kits were purchased from Jiangsu Meimian Bioengineering Institute (Jiangsu, China). The Estradiol (E2) ELISA kit was purchased from Beifang Bioengineering Institute (Beijing, China).

#### 5.1.2 Cell Culture

The human granulosa cell line (KGN) was maintained in DMEM/F12 supplemented with 10% FBS. Transfections were performed using Lipofectamine 3000. The mouse spermatogonia cell line (GC‐1) was treated with varying concentrations of caffeine (0–100 nM) or corticosterone (0–500 nM) for 24 h to assess *Gnas* expression and GR‐DNMT3B protein levels.

### 5.2 Animal Models and Treatment

#### 5.2.1 Ethical Statement

Specific pathogen‐free (SPF) Wistar rats (females: 200–240 *g*, males: 240–280 *g*) were purchased from the Experimental Center of the Hubei Medical Scientific Academy (No. 42000600042732; 42000600041821, Wuhan, China). All animal experimental procedures followed ethical norms and were approved by the Wuhan University Animal Experiment Center (Approval No. WHEF‐2022‐0286)

#### 5.2.2 PPCE Model (F0)

Male Wistar rats (F0) were administered caffeine (15, 30, or 60 mg/kg·d) via oral gavage for 8 weeks to cover the full spermatogenesis cycle. These doses were selected to establish a biologically interpretable paternal preconception exposure model rather than to define a clinical risk threshold. Controls received normal saline. F0 males were then mated with untreated females (1:2 ratio) to produce the F1 generation.

#### 5.2.3 Offspring Tracking (F1 & F2)

F1 offspring were standardized to 12 pups per litter. Developmental milestones, including vaginal opening and estrous cycles, were monitored from postnatal week 4 (PW4). To evaluate paternal‐line cross‐generational persistence, F1 males were mated with untreated females to produce the F2 generation.

#### 5.2.4 Intervention Studies

For GR antagonism, F0 males received RU486 (1 mg/kg·d). For ovarian intervention, PW4 F1 females received bilateral ovarian microinjections of 20 *µ*L AAV9‐*Gnas* or sh‐*Gnas* (2 × 10^13^ GC/mL).

### 5.3 In Vitro Fertilization (IVF) and Embryo Culture

To isolate the effect of sperm epigenetic information, C57BL/6 mice were treated with caffeine (120 mg/kg·d) for 8 weeks. Capacitated sperm from CON or PPCE males were used to fertilize mature oocytes from untreated females in KSOM medium. Resulting embryos were cultured for 48 h and transferred to pseudopregnant recipients. Fetal ovaries were collected at GD19 for molecular analysis.

### 5.4 Human Plasma and Sperm Samples

A total of 77 reproductive‐aged men were recruited from the Reproductive Medicine Center of Zhongnan Hospital. Among them, 69 subjects had valid paired measurements of both plasma cortisol concentration and sperm *Gnas*‐ICR methylation and were included in the primary correlation analysis. Samples with missing or technically invalid cortisol or methylation measurements were excluded from paired analyses. Reasons for exclusion included unavailable plasma cortisol data, insufficient sperm DNA, or failed methylation assay quality control. Plasma cortisol levels and sperm *Gnas*‐ICR methylation rates were measured following informed consent and institutional ethical approval (No. 2020188).

### 5.5 Molecular and Epigenetic Analysis

#### 5.5.1 DNA Methylation

Genomic DNA from sperm and ovaries was analyzed via bisulfite pyrosequencing and MethylTarget sequencing to determine CpG methylation at the *Gnas* imprinting control region (ICR). Genomic DNA was collected from F0 sperm and F1 offspring ovaries at GD20, PW6, and PW12 for *Gnas*‐ICR methylation analysis. Primer sequences and targeted CpG sites are listed in Table S5.

#### 5.5.2 Transcriptome Sequencing

RNA‐seq was performed on GD20 ovaries using the Illumina HiSeq platform. Differentially expressed genes (DEGs) were identified using a threshold of *p* < 0.05 and |*log_2_* fold change| > 1.

#### 5.5.3 Total RNA Extraction and Real‐Time Quantitative Polymerase Chain Reaction (RT‐qPCR)

Total RNA was extracted from ovarian tissue, testicular tissue, KGN cells, and GC‐1 cells using TRIzol Reagent and then converted into complementary DNA (cDNA). The RT‐qPCR assay was performed to quantify taget gene expression of specific DNA sequences using FastStart Universal SYBR Green Master Mix on the QuantStudio 5 RT‐qPCR cycler. Glyceraldehyde‐3‐phosphate dehydrogenase (GAPDH) was taken as an internal reference for the quantitative assay. The identified primers are listed in Table S4.

#### 5.5.4 ChIP‐qPCR

Chromatin immunoprecipitation (ChIP) was conducted in KGN cells using p‐CREB antibodies to quantify binding enrichment at the *StAR* promoter. In addition, ChIP assays were performed using paternal testis tissues with anti‐GR and anti‐DNMT3B antibodies to assess their respective enrichment at the *Gnas*‐ICR. The qPCR primers are listed in Table S5, and the antibodies involved are listed in Table S6.

Co‐immunoprecipitation (Co‐IP): Testicular protein lysates were prepared using ice‐cold IP lysis buffer supplemented with protease inhibitors. Equal amounts of protein were incubated with anti‐GR, anti‐DNMT3B, or normal rabbit IgG overnight at 4°C, followed by incubation with protein A/G agarose beads. Immunoprecipitates were washed, eluted, and analyzed by western blotting to detect GR–DNMT3B interaction. The antibodies used for Co‐IP are listed in Table S6.

#### 5.5.5 Protein Analysis

Western blotting and immunofluorescence (IF) were used to quantify protein expression (GR, DNMT3B, GNAS, StAR) and localization in testis, sperm, and ovarian tissues. The primary antibodies involved are listed in Table S6.

### 5.6 Statistical Analysis

Data are presented as Mean ± S.E.M. Statistical significance was determined using Student's *t*‐test for two‐group comparisons or one‐way ANOVA followed by Tukey's posthoc test for multiple groups. For the human exploratory analysis, the primary association between plasma cortisol concentration and sperm *Gnas*‐ICR methylation was assessed using Pearson correlation/regression based on all valid paired samples (*n* = 69). The primary scatter plot retained all valid paired samples without exclusion. Influential observations were evaluated using Cook's distance in SPSS Statistics, with 4/n used as the threshold. Sensitivity analyses included Pearson regression after exclusion of influential observations identified by Cook's distance, ROUT‐based outlier analysis performed in GraphPad Prism with the false discovery rate Q set to 1%, nonparametric Spearman correlation, and stratified analyses according to plasma cortisol concentration. ROUT analysis was used only as a sensitivity analysis and not for defining the primary dataset. Multiple linear regression was performed as an exploratory adjusted analysis to evaluate the association between plasma cortisol concentration and sperm *Gnas*‐ICR methylation after adjustment for age, BMI, serum testosterone level, and smoking status. *p* < 0.05 was considered statistically significant.

## Author Contributions

The study was designed by JH and HW. JH and LC performed most experiments and data analyses. JH performed animal procedures. TCW and YTL participated in human sample collection and related data acquisition. JH and LC participated in experimental design, data interpretation, and manuscript revision. JH and HW analyzed the data and revised the manuscript. The manuscript was written by JH and LC, and all authors read and edited the manuscript.

## Funding

This work was supported by grants from the National Natural Science Foundation of China (No. U23A20407, 82504938), and the Natural Science Foundation of Hubei Province (No. 2024AFB781).

## Consent

*Consent to Participate declaration*: The human exploratory study was approved by the Ethics Committee of Zhongnan Hospital of Wuhan University (Approval No. 2020188). All human participants provided written informed consent prior to enrollment, and before sample collection, explicitly authorizing the use of their clinical biospecimens and associated data for research purposes. The research process adhered to the principles of the Declaration of Helsinki.

## Conflicts of Interest

The authors declare no conflicts of interest.

## Supporting information




**Supporting File**: advs76434‐sup‐0001‐SuppMat.docx.

## Data Availability

All datasets generated and analyzed in this study are available from the corresponding author upon reasonable request.
