# Supplementary material for: Paternal Sperm Gnas‐ICR Epigenetic Programming Contributes to PPP‐Like Phenotypes in Female Offspring
Source: Adv Sci (Weinh). 2026 Jul 28:e76434. Online ahead of print. doi: 10.1002/advs.76434 (PMC13410812; doi:10.1002/advs.76434)
Supplement: Supplementary file 1 — Supporting File: advs76434‐sup‐0001‐SuppMat.docx. [file ADVS-9999-e76434-s001.docx]

**Supplementary Figures**

**Supplementary Fig. 1**


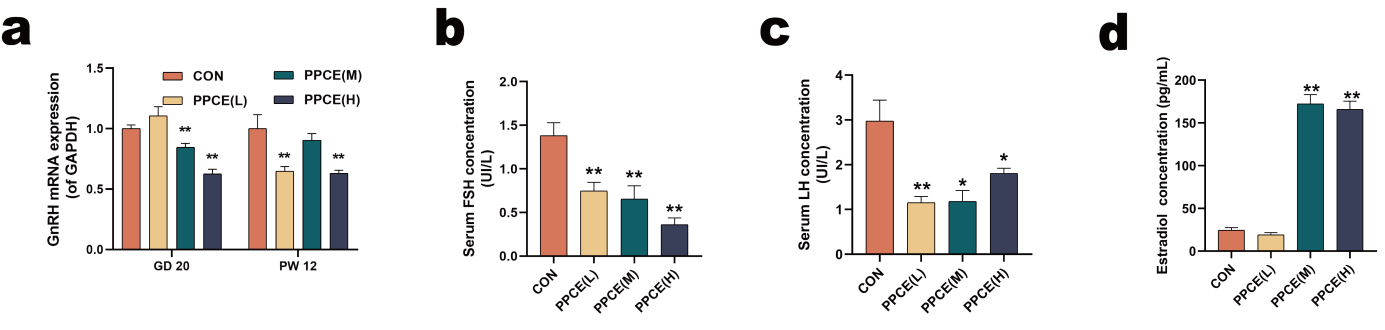


**Supplementary Fig. 1 | PPCE alters the hypothalamic-pituitary-ovarian endocrine profile in female offspring.** **a,** *GnRH* mRNA expression levels at GD20 and PW12, *n*=12. **b-d,** Serum FSH, LH, and E2 levels at PW12, *n*=12. Data are presented as mean ± S.E.M. ^*^*P*<0.05, ^**^*P*<0.01 *vs.* CON group. HPO: hypothalamic-pituitary-ovarian; PPCE: paternal preconception caffeine exposure; GD: gestational day; PW: postnatal week; GnRH: gonadotropin-releasing hormone; FSH: follicle-stimulating hormone; LH: luteinizing hormone; E2: estradiol; CON: control.

**Supplementary Fig. 2**


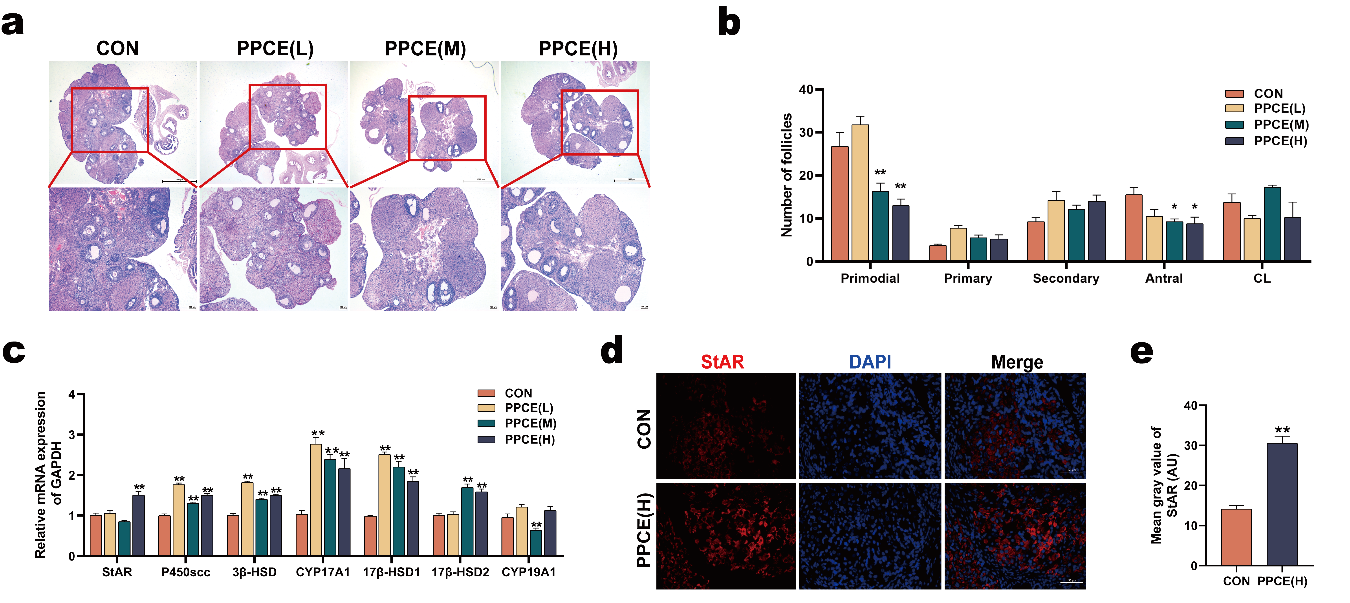


**Supplementary Fig. 2 | PPCE induces abnormal ovarian morphology and enhanced steroidogenic activity at PW12.** **a,** Representative ovarian H&E staining at PW12 (20× and 40×). **b,** Follicle counts at PW12, *n*=5. **c,** Ovarian mRNA expression levels of *StAR, P450scc, 3β-HSD1, CYP17A1, 17β-HSD1,* *17β-HSD2,* and *CYP19A1* at PW12, *n*=12. **d,e,** Representative StAR immunofluorescence images and quantification at PW12 (400×), *n*=5. Data are presented as mean ± S.E.M. ^*^*P*<0.05, ^**^*P*<0.01 *vs.* CON group. PW: postnatal week; PPCE: paternal preconception caffeine exposure; H&E: hematoxylin and eosin; StAR: steroidogenic acute regulatory protein; P450scc: cholesterol side-chain cleavage enzyme; 3β-HSD1: 3β-hydroxysteroid dehydrogenase 1; CYP17A1: cytochrome P450 family 17 subfamily A member 1; 17β-HSD1/2: 17β-hydroxysteroid dehydrogenase 1/2; CYP19A1: cytochrome P450 family 19 subfamily A member 1; IF: immunofluorescence; CON: control; CL: corpus luteum.

**Supplementary Fig. 3**


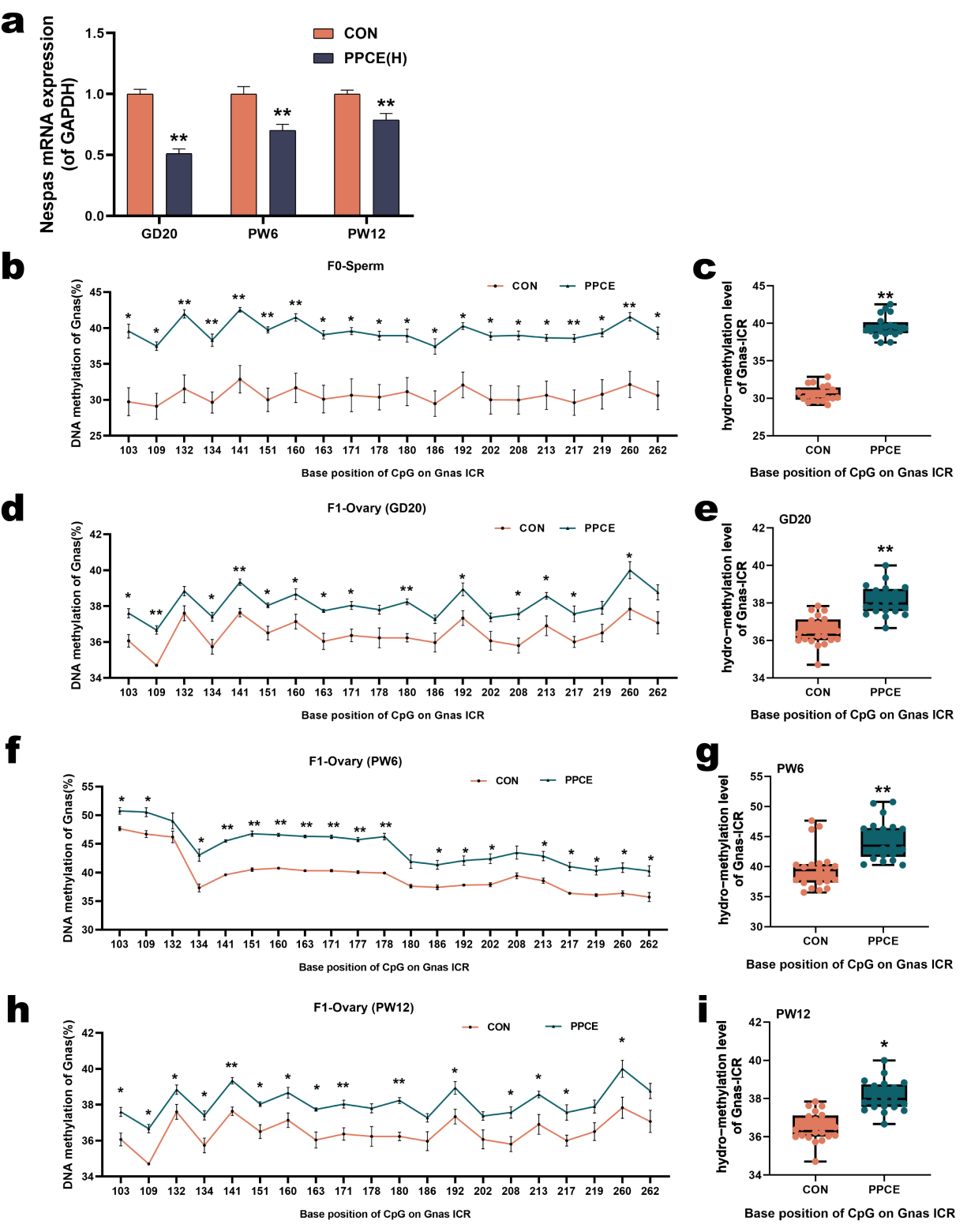


**Supplementary Fig. 3 | PPCE reduces ovarian Nespas expression and is associated with persistent Gnas-ICR hypermethylation across the examined developmental stages. a,** Nespas mRNA expression in F1 ovaries at GD20, PW6, and PW12, *n*=12. **b,c,** CpG-site-specific and average methylation levels of the Gnas-ICR in F0 sperm, *n*=3. **d,e,** CpG-site-specific and average methylation levels of the Gnas-ICR in F1 GD20 ovaries, *n*=3. **f,g,** CpG-site-specific and average methylation levels of the Gnas-ICR in F1 PW6 ovaries, *n*=3. **h,i,** CpG-site-specific and average methylation levels of the Gnas-ICR in F1 PW12 ovaries, *n*=3. Data are presented as mean ± S.E.M. ^*^*P*<0.05, ^**^*P*<0.01 *vs.* CON group. Nespas: GNAS antisense RNA 1; Gnas: GNAS complex locus; ICR: imprinting control region; PPCE: paternal preconception caffeine exposure; CON: control; GD: gestational day; PW: postnatal week.

**Supplementary Fig. 4**


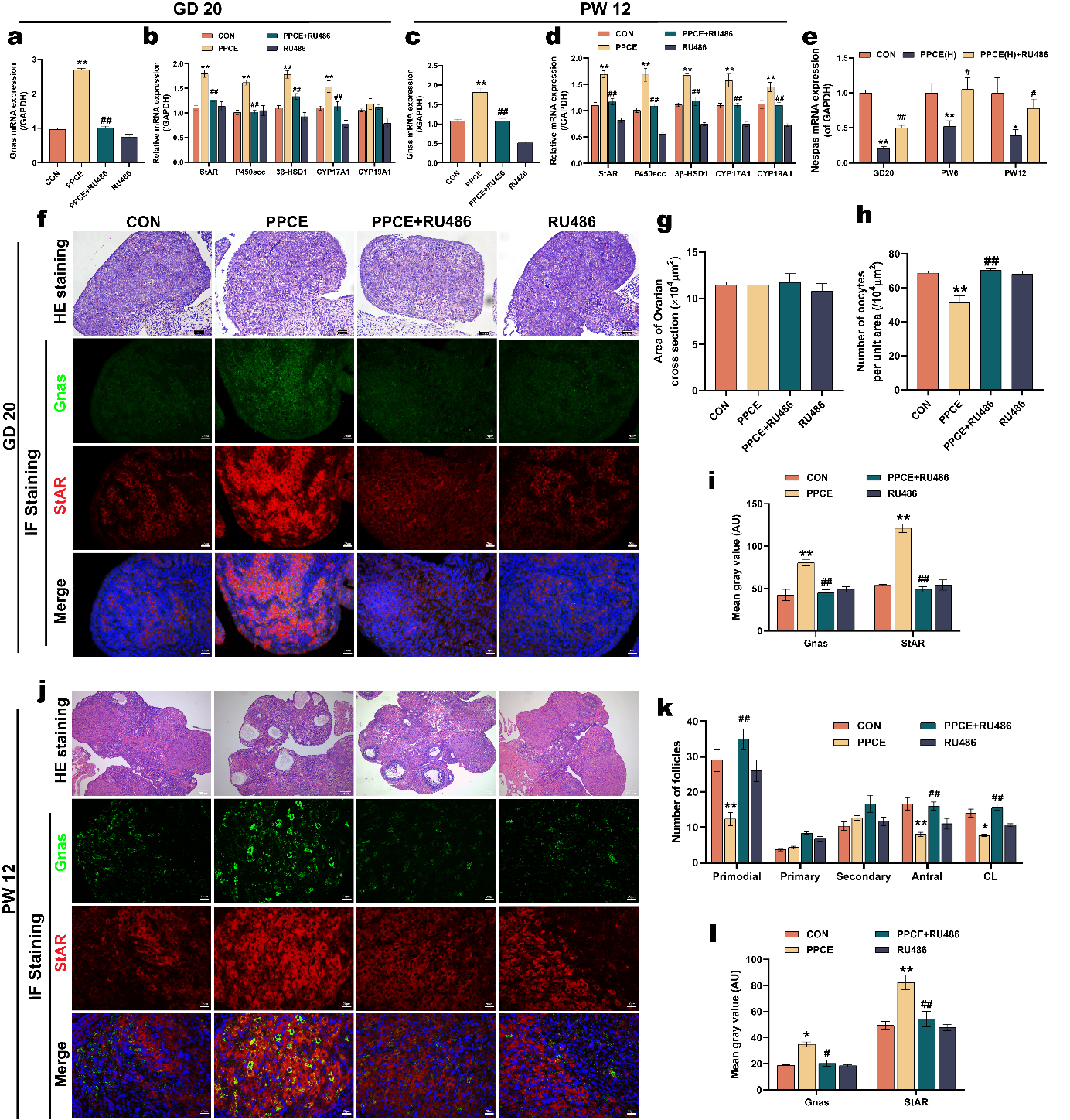


**Supplementary Fig. 4 | Paternal RU486 intervention attenuates PPCE-associated ovarian Gnas dysregulation and PPP-like phenotypes in female offspring.** **a,c,** Ovarian Gnas mRNA expression at GD20 and PW12, *n*=12. **b,d,** Ovarian *StAR, P450scc, 3β-HSD1, CYP17A1*, and *CYP19A1* mRNA expression at GD20 and PW12, *n*=12. **e,** Ovarian *Nespas* mRNA expression at GD20, PW6, and PW12, *n*=12. **f,** Representative H&E and GNAS/StAR IF staining at GD20. **g,h,** Ovarian cross-sectional area and oocyte number per unit area at GD20, *n*=5. **i,** GNAS and StAR IF quantification at GD20, *n*=5. **j,** Representative H&E and GNAS/StAR IF staining at PW12. **k,** Follicle counts at PW12, n = 5. **l,** GNAS and StAR IF quantification at PW12, *n*=5. Data are presented as mean ± S.E.M. ^*^*P*<0.05, ^**^*P*<0.01 *vs.* CON group. ^#^*P*<0.05, ^##^*P*<0.01 *vs.* PPCE group. RU486: mifepristone, a glucocorticoid receptor antagonist; PPCE: paternal preconception caffeine exposure; PPP: peripheral precocious puberty; GD: gestational day; PW: postnatal week; H&E: hematoxylin and eosin; IF: immunofluorescence; DAPI: 4′,6-diamidino-2-phenylindole.

**Supplementary Fig. 5**


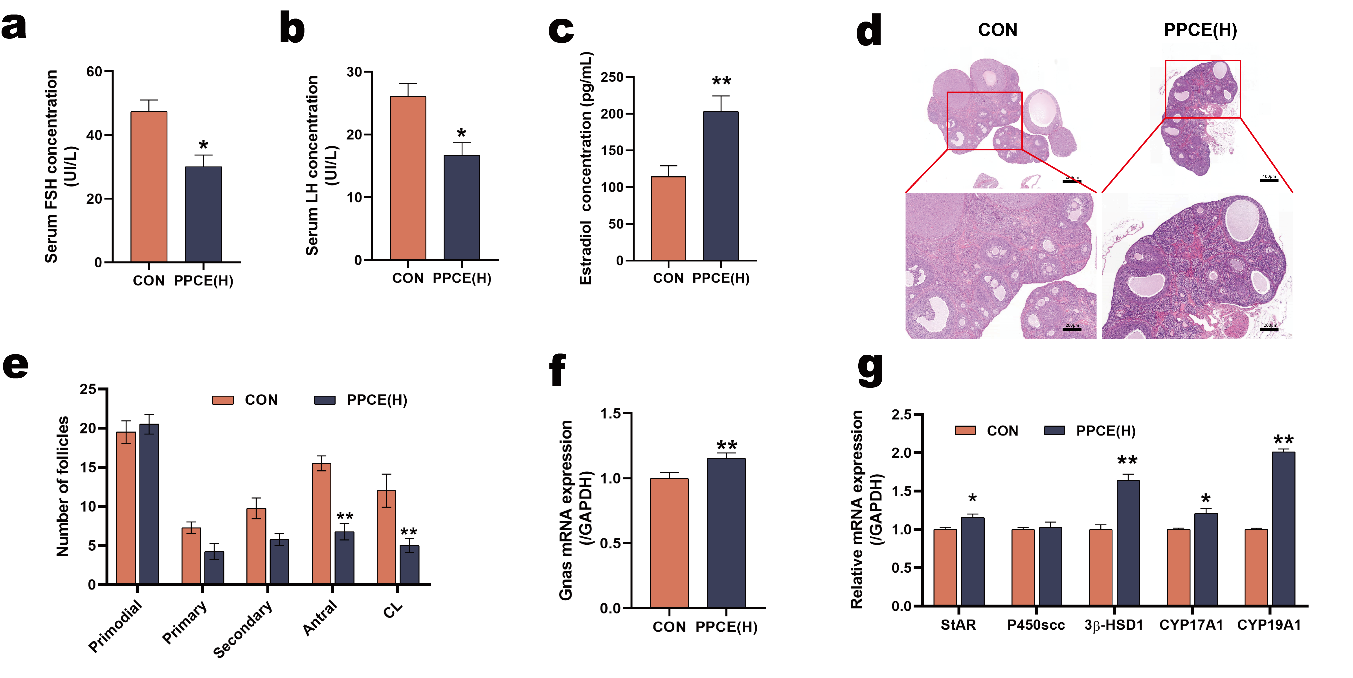


**Supplementary Fig. 5 | Paternal-line F2 offspring display persistent PPCE-associated ovarian endocrine alterations at PW12.** **a-c,** Serum FSH, LH, and E2 levels in F2 female offspring at PW12, *n*=12. **d,** Representative ovarian H&E staining at F2-PW12 (20× and 40×). **e,** Follicle counts at F2-PW12, *n*=5. **f,** Ovarian *Gnas* mRNA expression at F2-PW12, *n*=12. **g,** Ovarian mRNA expression levels of *StAR, P450scc, 3β-HSD1, CYP17A1*, and *CYP19A1* at F2-PW12, *n*=12. Data are presented as mean ± S.E.M. ^*^*P*<0.05, ^**^*P*<0.01 *vs.* CON group. PPCE: paternal preconception caffeine exposure; F2: second filial generation; PW: postnatal week; FSH: follicle-stimulating hormone; LH: luteinizing hormone; E2: estradiol; H&E: hematoxylin and eosin; Gnas: GNAS complex locus; StAR: steroidogenic acute regulatory protein; P450scc: cholesterol side-chain cleavage enzyme; 3β-HSD1: 3β-hydroxysteroid dehydrogenase 1; CYP17A1: cytochrome P450 family 17 subfamily A member 1; CYP19A1: cytochrome P450 family 19 subfamily A member 1; CON: control; CL: corpus luteum.

**Supplementary Tables**

**Table S1**. Sensitivity analyses of the association between plasma cortisol concentration and sperm Gnas-ICR methylation.

| **Analysis** | **Samples included** | **n** | **Statistical method** | **Effect estimates** | **P value** | **Interpretation** |
| --- | --- | --- | --- | --- | --- | --- |
| Primary analysis | All valid paired samples | 69 | Pearson regression | R²=0.1323 | 0.0021 | Weak positive association |
| Cook’s distance sensitivity analysis | Excluding 5 influential observations | 64 | Pearson regression | R²=0.09144 | 0.0152 | Nominal association remains |
| ROUT-based analysis | Excluding ROUT-defined outliers | 61 | Pearson regression | R²=0.002496 | 0.7022 | Not significant |
| Non-parametric analysis | All valid paired samples | 69 | Spearman correlation | r=0.1503 | 0.2177 | Not significant |
| Low-cortisol subgroup | Cortisol <400 μg/L | 58 | Pearson regression | R²=0.05497 | 0.0714 | Not significant |
| High-cortisol subgroup | Cortisol >400 μg/L | 11 | Pearson regression | R²=3.836e-005 | 0.9856 | Not significant |

**Table S2.** Exploratory multiple linear regression analysis of factors associated with sperm Gnas-ICR methylation level.

| **Variable** | **Estimate** | **Standard Error** | **95% CI** | **t value** | **P value** |
| --- | --- | --- | --- | --- | --- |
| Intercept | 0.004887 | 0.01265 | -0.02040 to 0.03017 | 0.3862 | 0.7006 |
| Cortisol concentration (μg/L) | 2.037×10^-5 | 5.918×10^-6 | 8.543×10^-6 to 3.220×10^-5 | 3.442 | 0.001 |
| Age (years) | 0.0004263 | 0.0002663 | -0.0001059 to 0.0009584 | 1.601 | 0.1144 |
| BMI | -0.0005806 | 0.0004208 | -0.001421 to 0.0002602 | 1.38 | 0.1725 |
| Testosterone (ng/mL) | 0.0007263 | 0.000819 | -0.0009102 to 0.002363 | 0.8869 | 0.3785 |
| Smoking status | 0.0004039 | 0.001546 | -0.002686 to 0.003494 | 0.2612 | 0.7948 |

Model R² = 0.1899.

**Table S3.** Primer sequences used for Gnas overexpression, Gnas knockdown, OE-Gnas-AAV, and sh-Gnas-AAV construction.

| **Gene** | **Sequences (5’ to 3’)** | |
| --- | --- | --- |
| *Hm-OE-Gnas* | F: CAACAGCGATGGTGAGAA | R:CATAGAATTCGGGAGGGAAG |
| *Hm-sh-Gnas* | F:CAAGGAGCAACAGCGAT | R:GGATGTAGTCCACTCTGAAC |
| *Rat-OE-Gnas-AAV* | F:AGGCCAACAAAAAGATCGAG | R:TCTCCGTTAAACCCATTAACA |
| *Rat-sh-Gnas-AAV* | F:GCCTCGGCAACAGTAAGA | R:ATCCTCATCTGCTTCACAATG |

**Table S4.** Primer sequences used for real-time quantitative PCR (RT-qPCR).

| **Gene** | **Sequences (5’ to 3’)** | |
| --- | --- | --- |
|  | **Forward** | **Reverse** |
| *Hm-GAPDH* | CATCATCCCTGCCTCTACTGG | GTGGGTGTCGCTGTTGAAGTC |
| *Hm-StAR* | GGCATCCTTAGCAACCAAGA | TCTCCTTGACATTGGGGTTC |
| *Hm-P450scc* | AGCGATTCATTGATGCCGATGCC | CTGGGTGTATATGTCAGCTTTA |
| *Hm-3βHSD1* | CTCTCCAGCATCTTCTG | TCACTACTTCCAGCAGG |
| *Hm-CYP17A1* | GTGGTTAAATGGACCCTG | CGAAGCACCTCTCGGA |
| *Hm-17βHSD1* | GGTTATGAGCAAGCCCTGAG | GGAAGCGGTTTGTGGAGAA |
| *Hm-17βHSD2* | AGCTTTAGCCGAATAGTTCGC | ATCGCAAAGATCAGTCGGAC |
| *Hm-CYP19A1* | CAGCCTGTCGTGGACTTGGT | GGCGATGTACTTTCCTGCACA |
| *Hm-Gnas* | CAGTACTTCCTGGACAAGATCGAC | TCAAACATGTGGAAGTTGACTTTG |
| *Rat-GAPDH* | GCAAGTTCAACGGCACAG | GCCAGTAGACTCCACGACA |
| *Rat-Gnas* | CCTGAAACAGTGCATATCAAGC | GAACTGCGTGTGTAGCTGAGTC |
| *Rat-StAR* | GGGAGATGCCTGAGCAAAGC | GCTGGCGAACTCTATCTGGGT |
| *Rat-Nespas* | ACTGATCCTCTCGTCTGGGA | TCCGCGCAACTTTATAGGGC |
| *Rat-P450scc* | GCCTTTTCTGGGCATAGTTG | GTGATGTGGGGACCAAGTTC |
| *Rat-3βHSD1* | CGGTGTGGATGACAACAGAG | AGACCAGAAACCAAGGAGGAA |
| *Rat-CYP17A1* | CTCTGCGTGGGTGTAATGAG | GTCGTCAATCTCTGGGCACT |
| *Rat-17βHSD1* | GGTTATGAGCAAGCCCTGAG | GGAAGCGGTTTGTGGAGAA |
| *Rat-17βHSD2* | AGCTTTAGCCGAATAGTTCGC | ATCGCAAAGATCAGTCGGAC |
| *Rat-CYP19A1* | CCCAGGAAGAGCGTGTTAGA | CATCAAGCAGCATTTGGACA |
| *Rat-GnRH* | TCTGCGAGGAGCTCTGG | GGGCCAGTGCATTACATCTT |
| *Rat-Dnmt3A* | CCGGGTGCTATCTCTCTCTTTG | TGACGATGGAGAGGTCATTG |
| *Rat-Dnmt3B* | GCAAGAGAGAGGCCCTCAG | TGTGAGGGAGATGCTCAGTG |
| *Rat-Dnmt3L* | GCTGGGCTTTGGGATTCTCT | CCCATCGGGATCTTGTCCAG |
| *Mus-GAPDH* | GCAAGTTCAATGGCACAG | GCCAGTAGACTCCACGACA |
| *Mus-StAR* | GGGAGATGCCTGAGCAAAGC | GCTGGCGAACTCTATCTGGGT |
| *Mus-P450scc* | GCTGCCTGGGATGTGATTTTC | GATGTTGGCCTGGATGTTCTTG |
| *Mus-3β-HSD1* | TCTACTGCAGCACAGTTGAC | ATACCCTTATTTTTGAGGGC |
| *Mus-CYP17A1* | ACTATCCGAGAAGTGCTGCGTAT | GCTCCGAAGGGCAAGTAACTC |
| *Mus-CYP19A1* | ATGGGCCTCCTTCTCCTGAT | CAGGCACTTCCAATCCCCAT |
| *Mus-Gnas* | GGTCTATCCGAGTGTACCCGA | GGCCTTCTCACTATCTCCGTTAAA |

**Table S5.** Primer sequences used for chromatin immunoprecipitation qPCR (ChIP-qPCR) and targeted bisulfite sequencing.

| **Gene** | **Sequences(5’ to 3’)** | |
| --- | --- | --- |
|  | **Forward** | **Reverse** |
| *Rat-Gnas-ICR* (ChIP-qPCR) | CAGCAAGCTCATCGACAAGC | GCACCACTTTTCTCCCTGGA |
| *Rat-Input* (ChIP-qPCR) | ACCTGGGGAAAACGTTGGTA | GAAGCCTTCTCATGTGGGGT |
| *Rat-Gnas-ICR*  (bisulfite sequencing) | GGTTTTYGAGAAGATTATAGTTTTTTATTTGAAG | ATATCRTATACRAAATTAAAAAATCTAACAACCTC |
| *Hm-Gnas-ICR*  (bisulfite sequencing) | TTTTTAATTTTAYGGGTAGTAGTTTTTGGATG | TTCCTCCTCAACTAAAAATCTCTCTA |

**Table S6.** Antibodies used for western blotting, immunofluorescence, ChIP-qPCR, and Co-IP analyses.

| **Protein** | **Antibody (catalog number)** | **Application** | **Dilution** |
| --- | --- | --- | --- |
| GNAS | Rabbit polyclonal(10150-2-AP) | IF# | 1:100 |
| StAR | Rabbit monoclonal (8449) | IF† | 1:100 |
| GR | Rabbit monoclonal (A19583) | IF, WB, ChIP, Co-IP* | IF (1:100); WB (1:1000); ChIP: 5 μg/IP; Co-IP: 5 μg/IP |
| DNMT3B | Rabbit polyclonal (A7239) | IF, WB, ChIP, Co-IP * | IF (1:100); WB (1:1000); ChIP: 3 μg/IP; Co-IP: 5 μg/IP |
| PKA-Cα | Rabbit polyclonal (4782) | WB† | 1:1000 |
| CREB | Rabbit monoclonal (9197) | WB† | 1:1000 |
| p-CREB | Rabbit monoclonal (9198) | WB, ChIP† | WB 1:1000; ChIP: 10 μg/IP |
| Normal rabbit IgG | Rabbit control IgG (AC005) | ChIP-qPCR, Co-IP control* | 5 μg/IP |
| GAPDH | Mouse monoclonal (AC002) | WB* | 1:5000 |

† Provided by Cell Signaling Technology Inc. (Danvers, MA).

# Provided by Proteintech (Wuhan, China)

* Provided by ABclonal (Wuhan, China)
